# Efficacy of Hematological and Coagulation Parameters in the Diagnosis and Prognosis of Patients with Acute Coronary Syndrome

**Published:** 2018-07

**Authors:** Arsalan Majeed Adam, Muhammad Armughan Ali, Arshad Ali Shah, Ather Hasan Rizvi, Aiman Rehan, Ansab Godil, Abdul Haseeb Abbas, Najam Ul Ain Durrani, Ali Tariq Shaikh, Muhammad Saad Ali Mallick, Muhammad Nawaz Lashari

**Affiliations:** 1 *Dow University of Health Sciences (DUHS), Karachi, Pakistan.*; 2 *Cardiology Department, Civil Hospital, Karachi, Pakistan.*

**Keywords:** *Acute coronary syndrome*, *Mean platelet volume*, *Erythrocyte indices*, *Leukocytes*

## Abstract

**Background** **:** The diagnosis and management of acute coronary syndrome (ACS) have improved significantly over the past few decades; however, the recognition of myocardial ischemia still proves to be a dilemma for cardiologists. The aim of this study was to determine the role of hematological and coagulation parameters in the diagnosis and prognosis of patients with ACS.

**Methods: **This prospective study recruited 250 patients with ACS and 250 healthy controls. The diagnostic role of hematological and coagulation parameters was assessed by comparing the patients with ACS with the control group. The relationships between these parameters and mortality were determined by dividing the patients into 2 groups: Group A (discharged) and Group B (patients who died within 30 days of follow-up). Multivariate Cox regression analysis was performed to calculate the hazard ratio (HR).

**Results: **The mean age of the patients was 55.14±10.71 years, and 65.2% of them were male. Prothrombin time (P<0.001), activated partial thromboplastin time (P<0.001), mean platelet volume (MPV) (P<0.001), white blood cell (WBC) count (P<0.001), and red blood cell distribution width (RDW) (P<0.001) were significantly higher in the case group than in the control group. WBC count (P<0.001), RDW (P<0.001), and MPV (P<0.001) were significantly higher in the controls than in the case group. The Cox regression model showed that RDW above 16.55% (HR=6.8), MPV greater than 11.25 fL (HR=2.6), and WBC higher than 10.55×10^3^/μL (HR=6.3) were the independent predictors of mortality.

**Conclusion**: In addition to being the independent predictors of short-term mortality, RDW, WBC, and MPV when used together with the coagulation profile may aid in the diagnosis of ACS in patients presenting with chest pain.

## Introduction

Despite great progress in the diagnosis and management of acute coronary syndrome (ACS), the recognition of myocardial ischemia still presents as a challenge to physicians, who continue to admit an inordinate number of patients, often over-diagnosing myocardial ischemia in low-risk patients. 

As was stated in a previous study, only one-fifth of the patients presenting with the complaint of acute chest pain required immediate treatment and hospital admission. However, a lack of a proper approach to patient stratification at the time of presentation leads to physicians’ over-admitting such patients.^1^ This over-evaluation burns hospital resources and threatens competence, especially in the low- and middle-income countries like Pakistan, where there is already a scarcity of resources.

Therefore, easily available laboratory tests which can be successfully used to reduce emergency department over-evaluation of patients with chest pain can have the potential to reduce hospital expenses. There is still a high incidence of mortality and complications among patients with ACS, and this burden has risen dramatically in the last 50 years and will continue to rise, as anticipated.^2^ The World Health Organization has predicted 11.1 million deaths from coronary heart disease in 2020.^3^ In view of all these alarmingly high numbers, it is important to stratify high-risk patients with ACS in order to determine when to employ more aggressive treatment strategies. 

Along with the existing guidelines which recommend cardiac troponin I (cTnI) and cardiac troponin T (cTnT) measurements as the preferred biochemical cardiac biomarkers for diagnosing ACS,^4^ recent studies have shown that raised levels of certain inflammatory markers including C-reactive protein, B-type natriuretic peptide, myeloperoxidase, placental growth factor, sCD40 ligand, ischemia-modified albumin, choline, and cystatin C are associated with an increased number of cardiovascular complications and a higher rate of mortality.^5^ Despite the usefulness of these novel biomarkers, a lack of routine availability, unorthodox laboratory kits and procedures, and high costs limit their worldwide utility. 

In the presence of these limitations, parameters which are easily measurable, cost-effective, and practicable by all healthcare professionals are sought to distinguish those patients who are at risk of death despite successful recanalization. Some of these quick and low-cost parameters reported in the previous literature are hemoglobin (Hb), red blood cell distribution width (RDW), mean platelet volume (MPV), and white blood cell (WBC) count.^6^

Nonetheless, there are conflicting data regarding the usability of these parameters as outcome predictors in patients with ACS, with some studies contradicting their outcome prediction potential. Moreover, there is no study assessing the prognostic value of these parameters in the Pakistani population.

The objectives of the present study were to determine whether on-admission hematological and coagulation parameters can play a role in the diagnosis of ACS and the prediction of short-term mortality (30 d) in this group of patients.

## Methods

This prospective cohort study was conducted at the Cardiology Department of Civil Hospital, Karachi, Pakistan. The study protocol was approved by the local ethics committee, and formal written informed consent was taken from all the patients. The study duration was 1 year, from January 2016 to January 2017, which also included a follow-up period of 1 month. 

A total of 400 patients who had been admitted to the cardiac emergency department with the chief complaint of new-onset chest pain were evaluated. From this total, 300 patients with the diagnosis of ACS were included in this study. Thirty patients refused to take part in the study, while 20 patients were excluded after considering the exclusion criteria. Also selected were 250 healthy controls with no history of cardiac disease and a normal electrocardiogram (ECG).

All patients with a complaint of chest pain (within the first 24 hours) and diagnosis of ACS at our cardiology emergency department who had a complete workup done (including complete blood count [CBC], cardiac enzymes, electrolytes, and coagulation profile) were included in our study. All patients with recent surgery, chronic inflammatory diseases, malignancy, active infection, and severe hepatic or renal impairment were excluded. Moreover, patients who had received anti-coagulant, anti-platelet, or anti-inflammatory drugs at least 7 days before blood sample collection were excluded. Age- and gender-matched control subjects were selected from among the patients who referred for a routine checkup to our outpatient department. 

For the purposes of the current study, ACS consisted of non-ST-segment elevation myocardial infarction (NSTEMI), defined by elevated cardiac ischemic markers (CK-MB or troponin) with typical chest pain and/or ECG changes indicating myocardial ischemia; ST-segment elevation myocardial infarction (STEMI), defined by typical chest pain lasting more than 20 minutes and associated with at least 1 of these characteristics: an increase greater than 1 ng/mL in troponin I and a new ST-segment elevation as measured from the J-point in 2 or more contiguous leads from leads V_1_, V_2_, and V_3_ measuring at least 0.2 mV or at least 0.1 mV in the remaining leads during the first 12 hours after symptom onset; and unstable angina, defined by typical chest pain and/or ECG changes indicating myocardial ischemia with negative cardiac ischemic markers.^7^

A complete physical examination and a detailed medical history of all the patients were taken at the time of admission. In addition, the Killip clinical examination classification and the New York Heart Association (NYHA) classification were evaluated.

An interviewer-based pilot-tested questionnaire was administered to every patient after 1 month following discharge to record the incidence of major adverse cardiac events and mortality.

The patients were divided into 2 groups: Group A was comprised of patients who were successfully discharged from the hospital and Group B was composed of patients who died during hospitalization or within 30 days of the follow-up period.

Blood samples were obtained within 30 minutes of admission. At baseline, venous blood samples were obtained to measure hematological indices, coagulation profile, and cardiac enzymes. An automated hematology analyzer, SYSMEX XN-1000, was used to measure the hematological indices. A blood coagulation analyzer, SYSMEX CA-1500, was employed to measure the coagulation profile. Cardiac enzymes (creatine kinase and creatine kinase-MB) were measured with a Roche Cobas c501 chemistry analyzer (Roche Diagnostics). Troponin T was measured via the highly sensitive assay (Roche Diagnostics). The number of diseased vessels was assessed through coronary angiography. Additionally, the left ventricular ejection fraction was evaluated using 2D echocardiography.

The categorical variables were compared using the χ^2^ test or the Fisher exact test, while the continuous variables were compared using the Mann-Whitney test or the independent-sample *t*-test. The normality was assessed by using the Shapiro-Wilk test. The receiver operating characteristics curve analysis was performed to determine the area under curve and the optimum cutoff of RDW, MPV, and WBC in predicting mortality. The area under curve with the ranges of 0.90 to 1, 0.80 to 0.90, 0.70 to 0.80, 0.60 to 0.70, and 0.50 to 0.60 was defined as excellent, good, fair, poor, and failing, respectively.^8^ Survival curves were made using the Kaplan-Meier analysis and compared via the log-rank test. All the variables whose p values were less than 0.25 in the univariate analysis were included in a multivariate Cox regression analysis. The backward stepwise likelihood ratio method was applied to identify the independent predictors of 30 days’ mortality. A 2-tailed p value of less than 0.05 was considered statistically significant.

## Results

The mean age of the study population was 55.14±10.71 years, and almost two-thirds (n=163, 65.2%) were male. More than half of the patients (n=176, 70.4%) were hypertensive, while almost one-third were known diabetics (n=85, 34%). The majority of the patients presented with either unstable angina (n=85, 34.0%) or STEMI (n=99, 39.6%). Out of the total population, 216 (86.4%) patients were successfully discharged from the hospital, while 34 (13.6%) died during the hospitalization or within 30 days of the follow-up period. 

Comparisons of the hematological and coagulation parameters in the control and ACS groups are depicted in Table 1. Group A patients were found to have significantly higher levels of prothrombin time (PT) (P<0.001), activated partial thromboplastin time (aPTT) (P<0.001), international normalized ratio (INR) (P<0.001), MPV (P<0.001), WBC (P<0.001), neutrophils (P<0.001), lymphocytes (P<0.001), and RDW (P<0.001) than Group B. On the other hand, the platelet count (P<0.001), Hb (P<0.001), red blood cell count (P<0.001), and hematocrit (P=0.002) were found to be higher in the control group. 

Table 2 shows a comparison of the baseline demographical, clinical, laboratory, and follow-up data between Group A and Group B. On comparison between Group A (n=216, 86.4%) and Group B (n=34, 13.6%), there was no significant difference in the prevalence of comorbid conditions such as diabetes (P=0.827) and hypertension (P=0.705). However, there was a greater percentage of patients with a family history of coronary artery disease in Group A than in Group B (P=0.013). The percentages of the left ventricular ejection fraction were found to be lower in Group B than in Group A (P=0.001). The duration of hospitalization of Group B was longer than that of Group A (P=0.002). 

The incidence rates of major adverse cardiac events on follow-up such as myocardial infarction (MI) (P=0.006), cardiogenic shock (P=0.008), rehospitalization (P=0.002), and coronary artery bypass grafting (P=0.003) were significantly higher in Group B than in Group A (Table 2).

Among the laboratory values, WBC count (P<0.001), RDW (P<0.001), and MPV (P<0.001) were significantly higher in the control group, whereas the platelet count (P=0.005) was significantly lower for the same group of patients. Moreover, the values of the coagulation profile such as PT (P=0.827), aPTT (P=0.874), and INR (P=0.693) were similar between the 2 groups. The values of the other biochemical markers are depicted in Table 2.

**Table 1:** Comparisons of the hematological and coagulation parameters between the control group and the group of patients suffering from acute coronary syndrome*

	Control (n=250)	Acute Coronary Syndrome (n=250)	^ₐ^P
Coagulation profile			
PT (s)	11.35 (1.80)	14.0 (4.80)	<0.001
aPTT (s)	27.76 (6.79)	35.5 (12.00)	<0.001
INR	1.06 (0.18)	1.25 (0.52)	<0.001
CBC profile			
Hemoglobin (g/dL)	13.28±0.84	12.48±1.62	<0.001
White blood cell count (×10^3^/μL)	7.69±1.60	10.48±3.24	<0.001
Neutrophils (×10^3^/μL)	6.31±0.67	7.40±0.95	<0.001
Lymphocytes (×10^3^/μL)	2.09±0.83	3.20±0.68	<0.001
Red blood cell count (×10^6^/μL)	4.71±0.34	4.27±0.58	<0.001
Hematocrit (%)	39.43±2.94	38.09±6.04	0.002
RDW (%)	14.4 (1.16)	15.9 (2.84)	<0.001
Platelet count (×10^3^/μL)	314.84±87.23	259.68±65.61	<0.001
MPV (fL)	10.74±0.65	11.14±1.37	<0.001

PT, Prothrombin time; aPTT, Activated partial thromboplastin time; INR, International normalized ratio; RDW, Red blood cell distribution width; MPV, Mean platelet volume

* Data are presented as means±SD or median (interquartile range).

**Table 2:** Comparisons of the baseline demographical, clinical, laboratory, and follow-up data between the patients who died and those who were discharged*

	Discharged (n=216) (Group A)	Mortality (n=34) (Group B)	^ₐ^P
Age (y)	55.38+10.60	53.62+11.42	0.375
Male	140 (64.8)	23 (67.6)	0.747
Previous history			
Smoking	62 (28.7)	11 (32.4)	0.664
Diabetes mellitus	74 (34.3)	11 (32.4)	0.827
Hypertension	153 (70.8)	23 (67.6)	0.705
Family history of CAD	93 (43.1)	7 (20.6)	0.013
Admission heart rate (bpm)	82.89+15.15	82.28+15.67	0.829
Admission SBP (mm Hg)	129.17+28.65	131.18+34.18	0.712
Admission DBP (mm Hg)	80.97+16.48	79.41+16.50	0.609
LVEF (%)	46.78+11.42	40.88+8.57	0.001
Duration of hospitalization (d)	7.00 (5.00)	9.00 (7.30)	0.002
Diagnosis			0.581
NSTEMI	59 (27.3)	7 (20.6)	
STEMI	83 (38.4)	16 (47.1)	
UA	74 (34.3)	11 (32.4)	
Killip class on presentation			0.024
≤I	139 (64.4)	15 (44.1)	
>I	77 (35.6)	19 (55.9)	
NYHA classification			0.043
≤I	76 (35.2)	6 (17.6)	
>I	140 (64.8)	28 (82.4)	
Number of diseased vessels			0.747
1 vessel	76 (35.2)	11 (32.4)	
>1 vessel	140 (64.8)	23 (67.6)	
Coagulation profile			
PT (s)**	14.00 (4.80)	13.0 (5.50)	0.827
aPTT (s) **	35.50 (12.38)	35.55 (12.05)	0.874
INR**	1.25 (0.52)	1.24 (0.46)	0.693
CBC profile			
Hemoglobin (g/dL)	12.38+1.44	13.12+2.41	0.088
White blood cell count (×10^3^/μL)	10.03+2.79	13.33+4.33	<0.001
Neutrophils (×10^3^/μL)	7.37+0.95	7.59+0.98	0.196
Lymphocytes (×10^3^/μL)	3.21+0.69	3.17+0.63	0.745
Red blood cell count (×10^6^/μL)	4.28+0.58	4.20+0.57	0.478
Hematocrit (%)	37.83+5.77	39.72+7.41	0.162
RDW (%)**	15.70 (2.70)	17.20 (2.89)	<0.001
Platelet count (×10^3^/μL)	264+62.36	230.15+78.15	0.005
MPV (fL)	11.01+1.17	12.01+2.06	<0.001
Cardiac enzymes**			
Troponin T**	142 (65.7)	23 (67.6)	0.827
CKMB**	48.0 (41.0)	45.91 (58.5)	0.928
CK	190.25 (268.8)	181.25 (408.8)	0.732
Follow-up			
Rehospitalization	35 (16.2)	13 (38.2)	0.002
MI	34 (15.7)	12 (35.3)	0.006
Cardiogenic shock	16 (7.4)	8 (23.5)	0.008
Stroke	15 (6.9)	4 (11.8)	0.304
Dialysis	4 (1.9)	0 (0)	0.555
GI bleeding	17 (7.9)	2 (5.9)	0.507
Transfusion	23 (10.6)	6 (17.6)	0.250
CABG	17 (7.9)	9 (26.5)	0.003
Stent placement	62 (28.7)	11 (32.4)	0.664
Access-site complication	1 (0.5)	1 (2.9)	0.254

CAD, Coronary artery disease; SBP, Systolic blood pressure; DBP, Diastolic blood pressure; LVEF, Left ventricular ejection fraction; NSTEMI, Non ST-elevation myocardial infarction; STEMI, ST-elevation myocardial infarction; UA, Unstable angina; NYHA, New York Heart Association; PT, Prothrombin time; aPTT, Activated partial thromboplastin time; INR, International normalized ratio; CBC, Complete blood count; RDW, Red blood cell distribution width; MPV, Mean platelet volume; CKMB, Creatine kinase-MB CK, Creatine kinase; MI, Myocardial infarction; GI, Gastrointestinal bleeding; CABG, Coronary artery bypass grafting

* Data are presented as means±SD, median (interquartile range)

** , or n (%).

**Table 3:** Univariate Cox regression analysis identifying the variables with p values less than 0.25

	HR	95% CI	P
Age	0.986	0.955-1.018	0.374
Male	1.112	0.542-2.282	0.772
Previous history			
Smoking	1.157	0.564-2.374	0.690
Diabetes mellitus	0.943	0.460-1.934	0.872
Hypertension	0.878	0.428-1.800	0.722
Admission heart rate	0.998	0.976-1.021	0.861
Admission SBP	0.995	0.975-1.016	0.642
Admission DBP	1.003	0.991-1.014	0.665
LVEF	0.958	0.929-0.988	0.006
Duration of hospitalization (d)	1.085	1.025-1.148	0.005
Killip>1	2.147	1.091-4.226	0.027
NYHA>1	2.387	0.988-5.764	0.053
NODV>1	1.123	0.548-2.305	0.751
Coagulation profile			
PT	0.975	0.898-1.058	0.539
aPTT	1.010	0.978-1.044	0.537
INR	0.885	0.385-2.034	0.774
CBC profile			
Hemoglobin	1.325	1.079-1.627	0.007
White blood cell count > 10.55 ×10^3^/μL	3.249	1.583-6.666	0.001
Neutrophils	1.270	0.889-1.816	0.189
Lymphocytes	0.914	0.559-1.494	0.719
Red blood cell count	0.835	0.483-1.443	0.518
Hematocrit	1.057	0.994-1.124	0.078
RDW>16.55 %	3.675	1.791-7.541	<0.001
Platelet count	0.993	0.989-0.998	0.005
MPV>11.25 fL	2.832	1.401-5.723	0.004
Cardiac enzymes			
Troponin T	1.080	0.527-2.217	0.833
CKMB	1.001	0.992-1.010	0.885
CK	1.001	0.999-1.002	0.291
Rehospitalization	2.784	1.393-5.561	0.004
MI	2.683	1.327-5.424	0.006
Cardiogenic shock	3.512	1.589-7.763	0.002
Stroke	1.617	0.570-4.590	0.367
GI bleeding	0.736	0.176-3.070	0.674
Transfusion	1.705	0.706-4.119	0.235
CABG	3.354	1.565-7.190	0.002
Stent placement	1.160	0.565-2.379	0.686

SBP, Systolic blood pressure; DBP, Diastolic blood pressure; LVEF, Left ventricular ejection fraction; NYHA, New York Heart Association; NODV, Number of diseased vessel; PT, Prothrombin time; aPTT, Activated partial thromboplastin time; INR, International normalized ratio; CBC, Complete blood count; RDW, Red blood cell distribution width; MPV, Mean platelet volume; CKMB, Creatine kinase-MB; MI, Myocardial infarction; GI, Gastrointestinal bleeding; CABG, Coronary artery bypass grafting

**Table 4:** Multivariate Cox regression analysis identifying the independent predictors of mortality

	HR	95% CI	P
Hemoglobin +1 g/dL	1.281	1.078-1.522	0.005
Neutrophils +1×10^3^/μL	1.443	0.974-2.137	0.067
LVEF+1 %	0.958	0.921-0.997	0.033
MI	3.120	1.416-6.872	0.005
Killip>1	1.939	0.908-4.144	0.087
CABG	5.661	2.350-13.637	<0.001
RDW>16.55 %	6.285	2.751-14.359	<0.001
MPV>11.25 fL	2.569	1.200-5.498	0.015
WBC>10.55×10^3^/μL	6.277	2.706-14.564	<0.001

MI, Myocardial infarction; LVEF, Left ventricular ejection fraction; CABG, Coronary artery bypass grafting; RDW, Red blood cell distribution width; MPV, Mean platelet volume; WBC, White blood cell

**Figure 1:**
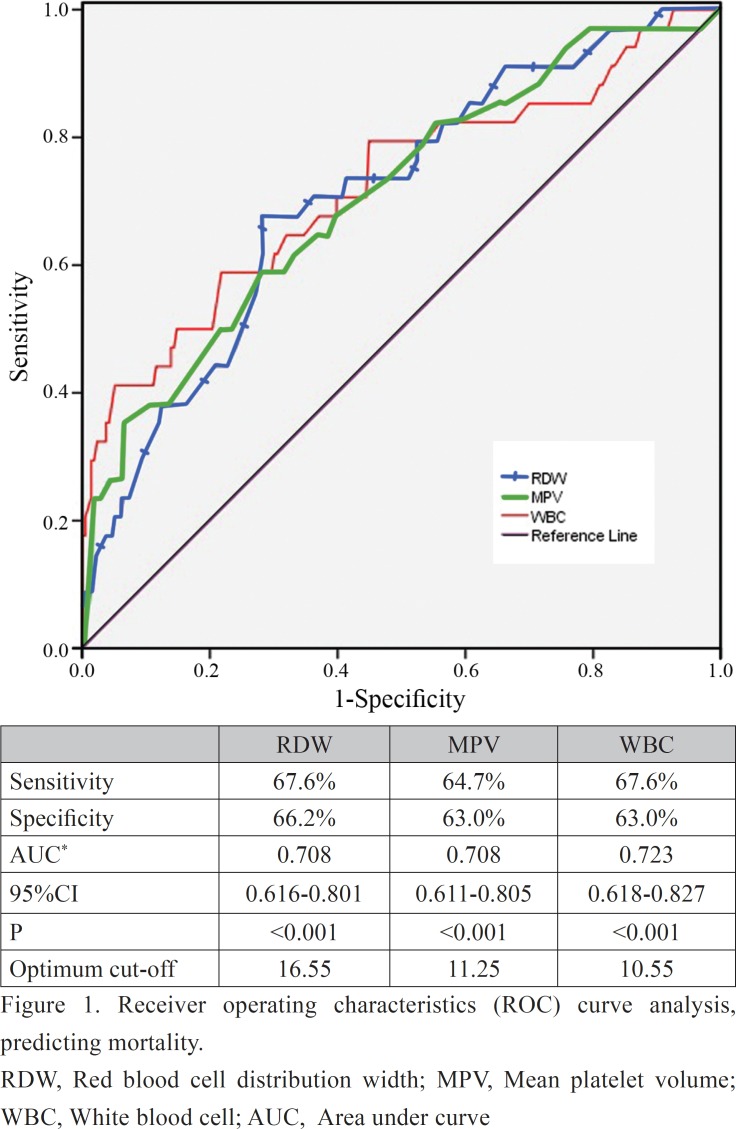
Receiver operating characteristics (ROC) curve analysis, predicting mortality.

RDW, Red blood cell distribution width; MPV, Mean platelet volume; WBC, White blood cell; AUC, Area under curve

The receiver operating characteristics curve analysis was performed to determine the discriminative ability of the hematological and coagulation parameters in predicting mortality. The area under curve of RDW, MPV, and WBC was 0.708, 0.708, and 0.723, respectively. RDW, MPV, and WBC were fair discriminants of mortality with cutoff values of 16.55% (sensitivity=67.6% and specificity=66.2%), 11.25 fL (sensitivity=64.7% and specificity=63.0%), and 10.55×10^^3^/μL (sensitivity=67.6% and specificity=63.0%), respectively (Figure 1).

**Figure 2:**
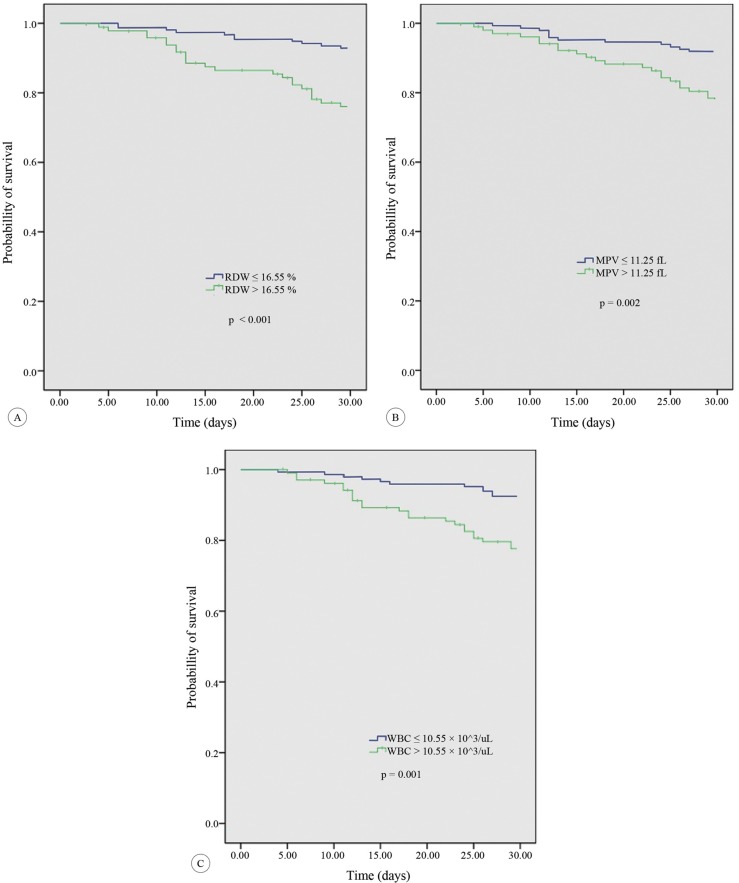
Kaplan–Meier survival curve for mortality in the patients with (A) RDW > 16.55%, (B) MPV > 11.25 fL, and (C) WBC > 10.5 103/µL.

RDW, Red blood cell distribution width; MPV, Mean platelet volume; WBC, White blood cell

The mortality rate in the follow-up period was significantly higher in the patients with RDW higher than 16.55% than in those with RDW equal to or less than 16.55% (24.0% [23/96] vs. 7.1% [11/154]; P<0.001). Similarly, the survival rates in the patients with MPV greater than 11.25fL were worse than those in the patients with MPV equal to or less than 11.25fL (21.6% [22/102] vs. 8.1% [12/148]; P=0.001). The patients with WBC greater than 10.55×10^^3^/μL were also associated with a high mortality rate as compared with those with WBC equal to or less than 10.55×10^^3^/μL (22.3% [23/103] vs. 7.5% [11/147]; P=0.001) (Figure 2).

On univariate Cox regression analysis, the following variables had p values less than 0.25: MI during the follow-up, coronary artery bypass grafting during the follow-up, cardiogenic shock during the follow-up, blood transfusion during the follow-up, duration of hospitalization, rehospitalization, left ventricular ejection fraction, RDW greater than 16.55%, MPV higher than 11.25 fL, WBC above 10.55×10^3^/μL, Killip class above 1, NYHA functional class above 1, Hb, neutrophils, hematocrit, and platelet count. These variables were then entered into the multivariate model (Table 3).

Based on the last step of the backward stepwise multivariate analysis, having an incident of MI during the follow-up (hazard ratio [HR]=3.1, 95% confidence interval (CI): 1.4–6.9; P=0.005), undergoing coronary artery bypass grafting during the follow-up (HR=5.7, 95% CI: 2.4–13.6; P<0.001), RDW greater than 16.55% (HR=6.3, 95% CI: 2.8–14.4; P<0.001), MPV greater than 11.25 fL (HR=2.6, 95% CI: 1.2–5.5; P=0.015), and WBC higher than 10.55×10^^3^/μL (HR=6.3, 95% CI: 2.7-14.6; P≤0.001) were found to be the independent predictors of mortality. Moreover, Hb (HR=1.3, 95% CI: 1.1-1.5; P=0.005) and the left ventricular ejection fraction (HR=1.0, 95% CI: 0.9-1.0; P=0.033) were also associated with increased mortality (Table 4). 

## Discussion

This is the first study in Pakistan to put forth 3 indices as the independent predictors of short-term mortality namely RDW, WBC, and MPV. In the present study, we also found that parameters like WBCs, neutrophils, lymphocytes, RDW, MPV, PT, aPTT, and INR were significantly higher in the patients with ACS than in the control group.

WBCs have been previously reported to be higher among patients with unstable angina and acute MI.^9^ Higher baseline WBC counts have been reported to be associated with a greater extent of coronary involvement.^10^ Some evidence suggests that WBCs may directly contribute to thrombus formation, impaired perfusion, and reperfusion injury.^11^

Our findings revealed a significantly higher level of MPV and a significantly lower platelet count in the patients with ACS than in the controls. This inverse relationship between MPV and platelet count has been reported in the previous literature.^12^ Similarly, many studies have reported low platelet counts in patients with ACS.^13^ Chu et al.^14^ reported MPV to be an early and independent diagnostic marker of ACS in patients presenting with acute chest pain. Furthermore, in their meta-analysis, Chu SG et al.^15^ reported that raised MPV was independently associated with the incidence of acute MI, mortality following MI, and restenosis following coronary intervention. Our study is in agreement with the existing literature. It is thought that increased MPV leads to a prothrombotic state, which renders patients vulnerable to intracoronary thrombosis and thus leads to the ischemia of the myocardium.

Unlike the studies conducted by Yilmaz et al.^16^ and Khandekar et al.,^13^ our study showed a significant difference in Hb and Hct between the 2 groups. We found that our patients with ACS had a significantly lower Hb and Hct levels than our control group. These results are similar to those reported by a previous study conducted by Yaghoubi et al.^17^ Additionally, we observed significant differences in the levels of coagulation parameters such as PT, aPTT, and INR between our 2 groups. Differences in these coagulation parameters have also been demonstrated previously by Yaghoubi et al.^17^ Moreover, we found significant differences in RDW levels between our case and control groups, with RDW being on the higher side in the patients with ACS. 

The pathophysiology behind the increased levels of all these hematological markers in patients having suffered acute coronary events is yet to be established. Certain mechanisms have been proposed regarding a few parameters (e.g., WBC, MPV, and RDW), but none of them has gained worldwide acceptance. Further studies are warranted to understand the underlying mechanism associating the hematological indices and coagulation profile of the patients to the incidence of ACS and the development of complications in patients with established coronary artery disease.

To find out the true outcome prediction potential of hematological parameters, we conducted a multivariate analysis (Cox regression analysis), which showed that an elevated baseline RDW level was an independent risk factor for short-term mortality in the patients with ACS. Recent studies have demonstrated a significant association between raised RDW levels and mortality in patients with heart failure, stroke, or stable coronary disease as well as in the general population.^18^^, ^^19^ Uyarel et al.^20^ reported that an elevated baseline RDW level in the patients undergoing percutaneous coronary intervention for STEMI was associated with an increased risk of in-hospital and long-term mortality. Wang et al.^21^ recruited 1654 ACS patients in their study and concluded that RDW was an independent predictor of re-infarction and short-term adverse outcomes in these patients. Additionally, high RDW has been associated with thrombus burden, poor reperfusion, and severity of the coronary lesion. A meta-analysis conducted by Su et al.^22^ supported the hypothesis that raised levels of RDW were associated with an increased incidence of future cardiovascular events and mortality in patients with established coronary artery disease. 

RDW is a coefficient of variability in erythrocyte volume and size. In our study, adjustment for the potential confounding factors (including Hb as an index for anemia) did not remove the association between raised RDW levels and short-term mortality in the patients with ACS. The relationship between elevated RDW levels and increased risk of mortality in these patients is incompletely understood. It has been proposed that inflammation induces changes in erythrocyte maturation, resulting in the release of immature cells into the circulation and increased RDW.^23^ As was demonstrated in previous studies,^24^ neurohormonal and adrenergic activation might influence erythropoiesis, raising RDW levels and making patients more prone to adverse clinical outcomes such as re-infarction and cardiovascular death. 

MPV, as reported in the previous literature, was found to be an independent predictor of mortality in our patient population as well. MPV is a widely available parameter which gives information about the size and function of platelets. Platelets play a crucial role in the pathogenesis of atherosclerosis and thrombus formation after coronary plaque rupture.^25^ Larger platelets are considered metabolically and enzymatically more reactive than smaller ones. Larger and hyperactive platelets, with the inherent property of accelerated formation and propagation of intracoronary thrombus, lead to an increased occurrence of acute thrombotic events.^26^ Raised MPV has previously been reported to increase the risk of MI independently of the known cardiovascular risk factors such as hypertension, dyslipidemia, increased fibrinogen, and increased plasma viscosity.^27^ Martin et al.^26^ suggested that increased MPV might be an independent risk factor for the post-MI recurrence of coronary events and mortality. Other investigators have demonstrated in small observational studies that MPV is higher in patients with MI than in those with stable angina pectoris and in healthy controls, suggesting that MPV is a risk factor for the severity of coronary artery disease.^28^ On the other hand, some studies have suggested that there is no association between raised MPV and mortality in patients with coronary artery disease.^29^^, ^^30^ Lopez-Cuenca et al.^31^ reported that MPV was of poor independent prognostic significance at 6 months’ follow-up in the patients with NSTEMI. Azab et al.^32^ suggested that the MPV/platelet count ratio had a higher predictive value than MPV alone in predicting adverse events after NSTEMI. Our findings are in contrast with such studies which negate any association between MPV and ACS mortality.

The mechanisms for raised MPV are not entirely understood. This increased platelet volume among patients with ACS may be explained by several theories. One theory postulates that more immature and larger platelets are released into the circulation after the consumption of normal-sized platelets.^33^^, ^^34^ Another explanation is that in a few patients, raised platelet volume may be due to the presence of inherently large platelets. It is well-known that platelet count and MPV, among other hematological parameters, are generally inheritable. Three loci and 3 common single-nucleotide polymorphisms related to MPV accounting for about 5% variances in the MPV value in a population were identified by Meisinger et al.^35^ and Soranzo et al.^36^ in their genome-wide association study, which analyzed the relative risk posed by blood cell loci in the development of cardiovascular disease, particularly MI. On analyzing 22 genetic loci reproducibly associated with relevant hematological factors including red and WBC counts and platelet volume and count, 1 quantitative trait locus related to MPV, at 12q24, was found to be a risk locus for coronary artery disease. Apart from this locus, no evidence was found to be contributing to the risk of coronary artery disease or MI. The new locus associated with MPV may play a possible role in survival and prognosis among patients suffering from ACS. This possibility warrants further studies and investigation.

Raised WBC levels were found to be another independent predictor of 30 days’ mortality in our study. A number of epidemiological studies have supported the prognostic value of baseline WBCs as a predictor of death both in the short and long terms following ACS. Nunez et al.^37^ concluded that elevated levels of WBCs were associated with mortality in either type of MI (STEMI and NSTEMI), independently of the other known prognostic factors. In contrast to our findings, a study conducted by Huang et al.^38^ reported no statistically significant association between total WBC count and the short-term, midterm, and long-term occurrence of major cardiac adverse events and with the severity of coronary atherosclerosis in patients with ACS. Furthermore, they reported neutrophils to be of independent predictive value in patients suffering from ACS with adverse cardiac events, which also opposes our findings. In our study, we found that differential counts were of no prognostic significance.

Some of the proposed mechanisms which underlie the association between raised WBC levels and the development of complications and death include resistance to thrombolytic therapy due to alterations in the microcirculation,^39^ a no-reflow phenomenon caused by leukocytes,^40^ hypercoagulable state,^41^ indirect cardiotoxicity mediated by pro-inflammatory cytokines,^42^ ischemia/reperfusion injury,^43^ and expansion of the infarct size. Leucocytes play a central role in the inflammatory reparative response initiated to form a scar in place of a necrotic tissue following acute MI. This may suggest that the greater the size of the infarct, the higher the leukocyte response, an affirmation based on experimental studies which show a linear relationship between the amount of necrosis and the level of both the local and the systemic leukocyte response. As greater-sized infarcts are more prone to develop complications such as heart failure and death, this reflection of infarct size by WBC counts may have potential prognostic importance. 

It must be noted that our study has certain limitations. Firstly, the study sample represents a small population with more or less similar demographics and the subjects were recruited from only 1 tertiary care hospital in Karachi, which limits generalizability. This makes it imperative that larger scale studies, over a large geographical area including more subjects, be undertaken in order to adequately identify the diagnostic and prognostic role of these hematological parameters. Secondly, the hematological and coagulation parameters were from blood samples drawn only once at the time of admission and blood sampling time was not standardized.

## Conclusion

Hematological and coagulation parameters have a potential to be used as diagnostic and prognostic markers. Not only are these biochemical markers cost-effective and inexpensive, but also they are easily measured and hence can cater to all global healthcare facilities. Prompt and early stratification of high-risk patients will enable physicians to carefully monitor and manage such patients and schedule them for frequent follow-ups, playing a part in reducing mortality. This is also useful in developing countries like Pakistan, where healthcare facilities are already overburdened and a reduction in the admittance of low-risk patients will relieve this unnecessary economic burden. However, the conflicting results of some previous studies have made the usage of these markers controversial. More comprehensive studies with larger sample sizes are, therefore, needed to further validate our findings.

## Notes:

***This paper should be cited as: ***Adam AM, Ali MA, Shah AA, Rizvi AH, Rehan A, Godil A, Abbas AH, Durrani NUA, Shaikh AT, Ali Mallick MS, Lashari MN. Efficacy of Hematological and Coagulation Parameters in the Diagnosis and Prognosis of Patients with Acute Coronary Syndrome. J Teh Univ Heart Ctr 2018;13(3):115-125.
